# Supplementary material for: A universal electromagnetic energy conversion adapter based on a metamaterial absorber
Source: Sci Rep. 2014 Sep 9;4:6301. doi: 10.1038/srep06301 (PMC4158331; doi:10.1038/srep06301)
Supplement: Supplementary Information — SUPPLEMENTARY MATERIAL [file srep06301-s1.pdf]

A universal electromagnetic energy conversion adapter based on a metamaterial absorber

Authors: Yunsong Xie[1], Xin Fan[1], Jeffrey D. Wilson[2], Rainee N Simons[2], Yunpeng Chen[1], and John Q. Xiao[1]*

Affiliations:

[1]Department of Physics and Astronomy, University of Delaware, Newark, Delaware 19716 USA

[2]Glenn Research Center, National Aeronautics and Space Administration, Cleveland, Ohio 44135 USA

*Correspondence to: jqx@udel.edu

SUPPLEMENTARY MATERIAL

Simulation result of an ideal UEECA with zero loss materials

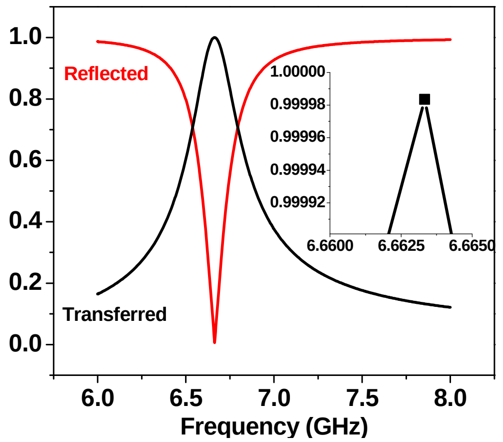

Figure S1. The Simulated S-parameters of the UEECA with zero loss material.

The purpose of this simulation is to test the signal transferring performance of UEECA in the ideal case, in which the copper is substituted by PEC and the loss tangent of Roger 5880LZ was eliminated. In this simulation, the structure shown in Fig. 2 (c) was used. All the dimensional parameters remained the same except that the thickness of air gap was changed from 0.8 mm to 0.7775 mm to obtain the optimum signal transfer ratio of 0.99998.

Measurement result of the UEECA constructed using FR4

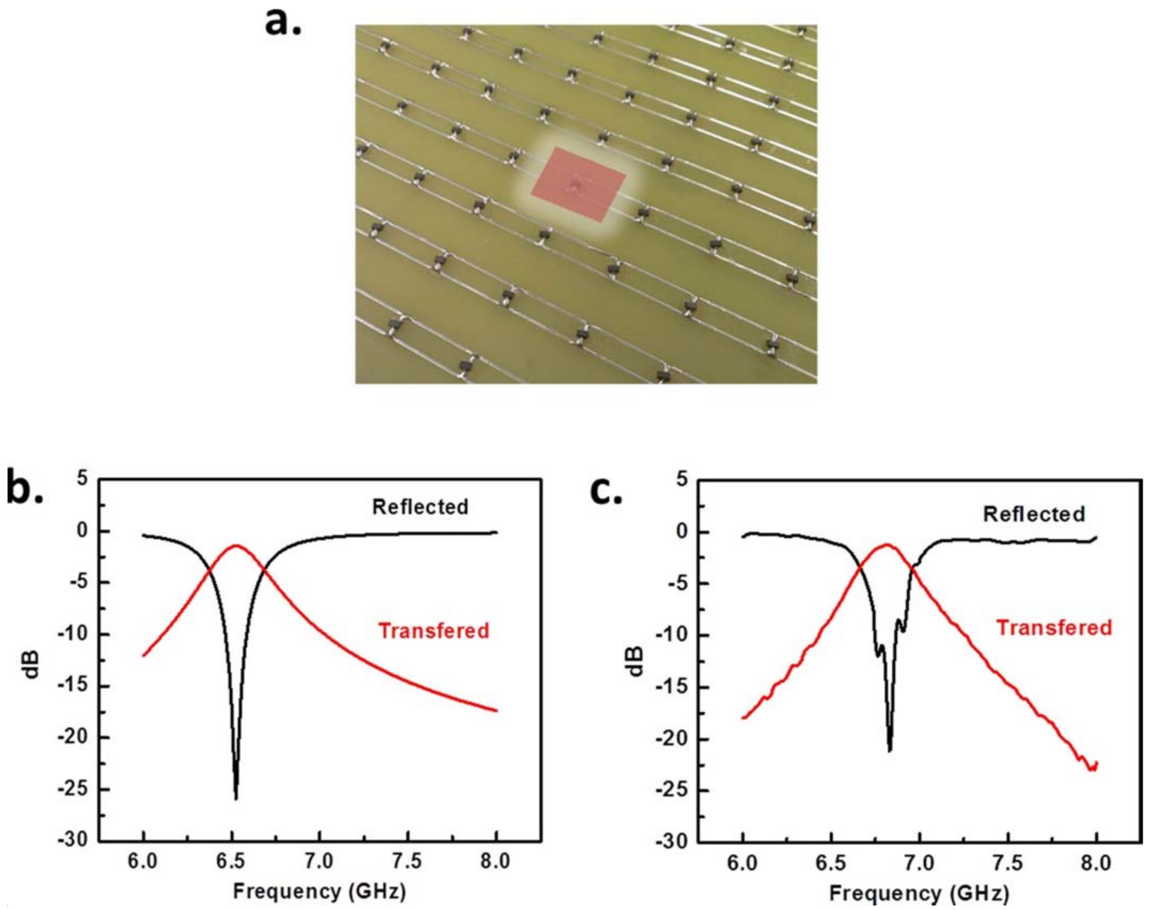

Figure.S2 (a) The picture of the UEECA constructed using FR4, the ratio of the reflected and transferred microwave in (b) simulation and (c) measurement.

FR-4 has been used to construct a demonstration of UEECA as shown in Fig. S2 (a). This array is formed with 12 by 12 units. An Avago HSMS-286B diode is attached to every unit of the UEECA. The dimensional parameters of the unit are a=15 mm, w=4 mm, s=0.5 mm, d=1 mm, g=2 mm, h=1.58 mm and t=1 mm. The real part and loss tangent of the permittivity of Rogers 5880LZ are set to be 4.4 and 0.02 respectively. A maximum signal transfer ratio of 87% (-1.2 dB) has been achieved in a simulation at frequency 6.5 GHz, where the reflection is -26 dB. The

experimental measurement of the sample gives maximum signal transfer ratio of 86% (-1.3 dB) at 6.8 GHz, where the reflection reads -23 dB.

Derivation of the transmission line model of the front structure of MA with the periodic boundary condition

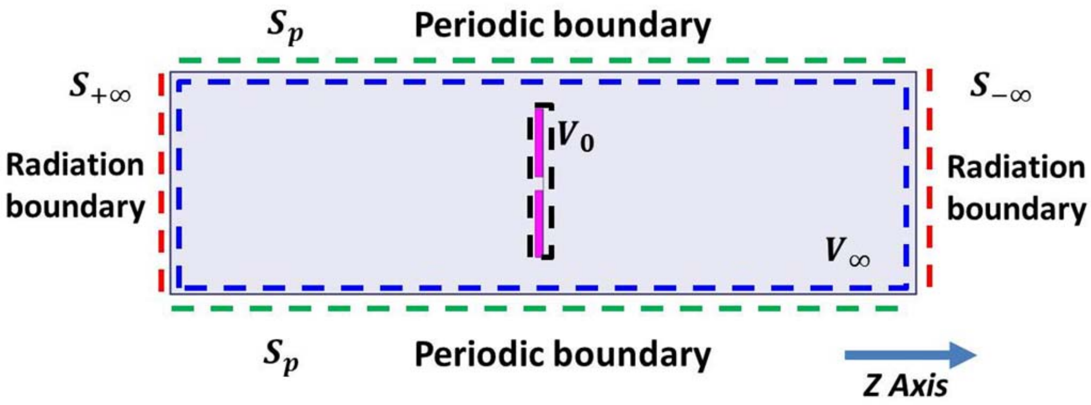

Figure.S3 A sketch of the structure with only the front structure as in Fig. 1 (a). The notations include the radiation boundary surfaces in the positive (negative) z axis $S_{+\infty}(S_{-\infty})$ indicated by the red dashed lines, periodic boundary surfaces S_p shown by the green dashed lines, far field volume V_∞ surrounded by the blue dashed line, and the volume containing only the antenna V_0 surrounded by the black dashed line.

The periodically distributed arbitrary front structure is considered as an antenna as shown in Fig. S3. A conceptual energy-converting sensor modeled by a load with impedance Z_L is attached to the front structure of every signal unit. The value of Z_L is:

$$Z_L = \frac{2P_L}{|I|^2} \qquad \text{(S1)}$$

where P_L is the converted EM power or the power being emitted from the sensor and I is the intensity of the current exciting the antenna. For an antenna with signal resonant, the rest of the components in Fig. 1 (c) can be expressed by[1]:

$$Z_A^T = \frac{2P^T}{|I|^2} \qquad \text{(S2)}$$

$$L_A = \frac{4\widetilde{W_m}}{|I|^2} \qquad \text{(S3)}$$

$$C_A = \frac{|I|^2}{4\omega^2\widetilde{W_e}} \qquad \text{(S4)}$$

The radiation loads in the positive (+) and negative (-) z axis directions can be expressed as:

$$Z_{A\pm}^{Rad} = \frac{2}{|I|^2}\frac{\left(P_{A+}^{Rad}+P_{A-}^{Rad}\right)^2}{P_{A\pm}^{Rad}} \qquad \text{(S5)}$$

where Z_A^T and $Z_{A\pm}^{Rad}$ are the impedances representing the thermal dissipation and radiation, and L_A and C_A are the inductive and capacitive components of the antenna. P^T, $P_{\pm}^{Rad}$, $\widetilde{W_m}$ and $\widetilde{W_e}$ are defined under the condition that the antenna is excited by a hypothetical current I. P^T represents the power dissipated and converted into thermal energy because of the non-zero imaginary part of either permittivity or permeability of the substrate and ohmic losses in the non-perfect metal conductor. $P_{\pm}^{Rad}$ is the power being radiated to the left (-) and right (+) sides of the structure, which are the same for an antenna of symmetric structure as shown in Fig. 1 (a) and Fig. S3. $\widetilde{W_m}$ and $\widetilde{W_e}$ stand for the stored magnetic and electric field power, respectively. According to the most general form for the Poynting theorem, the values of P^T, $P_{\pm}^{Rad}$, $\widetilde{W_m}$ and $\widetilde{W_e}$ can be calculated by[1-4]:

$$P^{T} = \frac{\omega}{2}\int_{V_\infty} \varepsilon''|\boldsymbol{E}|^2 + \mu''|\boldsymbol{H}|^2\, dv \qquad \text{(S6)}$$

$$P_{\pm}^{Rad} = Re\oint_{S_{\pm\infty}} (\boldsymbol{E}\times\boldsymbol{H}^*)\cdot d\boldsymbol{S} \qquad \text{(S7)}$$

$$\widetilde{W_m} = \frac{1}{2}\left[\int_{V_\infty}\frac{1}{4}(\varepsilon'|\boldsymbol{E}|^2 + \mu'|\boldsymbol{H}|^2)dv - \frac{r_\infty}{c}Re\oint_{S_{-\infty}+S_{+\infty}+S_{\boldsymbol{p}}}(\boldsymbol{E}\times\boldsymbol{H}^*)\cdot d\boldsymbol{S} + \frac{1}{2\omega}Im\oint_{S_{-\infty}+S_{+\infty}+S_{\boldsymbol{p}}}(\boldsymbol{E}\times\boldsymbol{H}^*)\cdot d\boldsymbol{S}\right] \qquad \text{(S8)}$$

$$\widetilde{W_e} = \frac{1}{2}\left[\int_{V_\infty}\frac{1}{4}(\varepsilon'|\boldsymbol{E}|^2 + \mu'|\boldsymbol{H}|^2)dv - \frac{r_\infty}{c}Re\oint_{S_{-\infty}+S_{+\infty}+S_{\boldsymbol{p}}}(\boldsymbol{E}\times\boldsymbol{H}^*)\cdot d\boldsymbol{S} - \frac{1}{2\omega}Im\oint_{S_{-\infty}+S_{+\infty}+S_{\boldsymbol{p}}}(\boldsymbol{E}\times\boldsymbol{H}^*)\cdot d\boldsymbol{S}\right] \qquad \text{(S9)}$$

where $\boldsymbol{E}$ and $\boldsymbol{H}$ are the electric field and magnetic field distributions in a single unit under the periodic boundary conditions which are obtained by strictly solving the given structure and material. ω is the angular frequency of the EM wave, and ε' (μ') and $\varepsilon''(\mu'')$ are the real and imaginary part of the permittivity (permeability) of the constituent material, respectively. V_0 is the volume that contains the antenna, V_∞ is the volume that covers the region, the edges of which can be viewed as in the far field region, c is the speed of light in vacuum, and r_∞ is the distance between the antenna and the edge of the V_∞ regime. It is reasonable to make the conclusion that there is no net energy flow through the periodic boundary surfaces:

$$\oint_{S_{\boldsymbol{p}}}(\boldsymbol{E}\times\boldsymbol{H}^*)\cdot d\boldsymbol{S} = \boldsymbol{0} \qquad \text{(S10)}$$

It is worth noticing that the equation above does not indicate that there is no coupling between the units. Instead the Poynting vectors in the perpendicular direction at the periodic boundaries cancel out, which results in a vanishing integral. The calculations of $\widetilde{W_m}$ and $\widetilde{W_e}$ should consider

the electric and magnetic power that is stored in the volume of the antenna structure. Therefore, the equations (8-9) can be expressed by:

$$\widetilde{W_m} = \frac{1}{2}\left[\int_{V_\infty} \frac{1}{4}(\varepsilon'|\boldsymbol{E}|^2 + \mu'|\boldsymbol{H}|^2)dv - \frac{r_\infty}{c} Re \oint_{S_{-\infty}+S_{+\infty}} (\boldsymbol{E} \times \boldsymbol{H}^*) \cdot d\boldsymbol{S} + \frac{1}{2\omega} Im \oint_{S_{-\infty}+S_{+\infty}} (\boldsymbol{E} \times \boldsymbol{H}^*) \cdot d\boldsymbol{S}\right] \quad \text{(S11)}$$

$$\widetilde{W_e} = \frac{1}{2}\left[\int_{V_\infty} \frac{1}{4}(\varepsilon'|\boldsymbol{E}|^2 + \mu'|\boldsymbol{H}|^2)dv - \frac{r_\infty}{c} Re \oint_{S_{-\infty}+S_{+\infty}} (\boldsymbol{E} \times \boldsymbol{H}^*) \cdot d\boldsymbol{S} - \frac{1}{2\omega} Im \oint_{S_{-\infty}+S_{+\infty}} (\boldsymbol{E} \times \boldsymbol{H}^*) \cdot d\boldsymbol{S}\right] \quad \text{(S12)}$$

The derivation above is based on the assumption that there is only one resonance in the frequency spectrum. Such a resonant structure can therefore be characterized by an RLC resonator. In a more general case, an antenna with complex response can be modeled by replacing the components L_A and C_A with X_A and the structure is characterized with only imaginary impedance:

$$X_A = j\frac{4\omega(\widetilde{W_m} - \widetilde{W_e})}{|I|^2} \quad \text{(S13)}$$

In both cases, a broadband absorption MA can be design when Eq. (1) is satisfied throughout the frequency range of interest.

References

1 Geyi, W., Jarmuszewski, F. & Qi, Y. H. The foster reactance theorem for antennas and radiation Q. *IEEE Trans. Antennas Propag.* **48**, 401-408, (2000).

2 Collin, R. E. & Rothschild, S. Evaluation of antenna Q. *IEEE Trans. Antennas Propag.* **Ap12**, 23-27 (1964).

3 Fante, R. L. Quality factor of general ideal antennas. *IEEE Trans. Antennas Propag.* **Ap17**, 151-155 (1969).

4 Rhodes, D. R. On stored energy of planar apertures. *IEEE Trans. Antennas Propag.* **Ap14**, 676-683 (1966).
